# Resolutions of Respect to Dr. George E. Nettleton

**Published:** 1905-11

**Authors:** 


### Obituary.

### RESOLUTIONS OF RESPECT TO DR. GEORGE E. NETTLETON.

New Haven, Conn., September 20, 1905.

Whereas, By the death of our friend and co-worker, Dr. George E. Nettleton, this Association records the loss of one through whose efforts, to a very great degree, we are indebted for our existence as an organized body, and while of an extremely retiring disposition, he was always on the alert in consideration of the Association's welfare; and

Whereas, Our profession loses a representative member, an earnest worker, who for more than thirty years had been honored and trusted by a large clientele. His positive yet affable nature won for him more than usual the respect and confidence of his patients. He was also a warm friend and helper to many young men entering our profession. During many years' association we never heard him speak a harsh or unkind word. His memory will always be cherished by this Association, never ceasing to mourn his loss; therefore, be it

*Resolved,* That we extend to the family of our departed brother our sincere sympathy in this hour of their bereavement; and be it further

*Resolved,* That these resolutions be spread upon the records of this Association, and a copy sent to his family.

E. S. Gaylord,
C. W. Strang,
H. A. Spang,
*Committee.*

Hartford, Conn., September 11, 1905.

Whereas, We, members of the Hartford Dental Society, have learned with profound sorrow of the death of Dr. George E. Nettleton, of New Haven; therefore be it

*Resolved,* That we deeply sympathize with his family and present our condolence to the New Haven Dental Association in the loss they have sustained. Honored and respected, he was for years regarded as one of the leading practitioners of dentistry in our State. We shall ever revere his memory and deplore his departure; and be it further

*Resolved,* That a copy of these resolutions be sent to his family, likewise to the New Haven Dental Association, and also that they be entered upon the records of this Society.

ALBERT E. CARY,
*Secretary.*

